# Early identification of acute kidney injury in Russell’s viper (*Daboia russelii*) envenoming using renal biomarkers

**DOI:** 10.1371/journal.pntd.0007486

**Published:** 2019-07-01

**Authors:** Indira Ratnayake, Fahim Mohamed, Nicholas A. Buckley, Indika B. Gawarammana, Dhammika M. Dissanayake, Umesh Chathuranga, Mahesh Munasinghe, Kalana Maduwage, Shaluka Jayamanne, Zoltan H. Endre, Geoffrey K. Isbister

**Affiliations:** 1 South Asian Clinical Toxicology Research Collaboration, Faculty of Medicine, University of Peradeniya, Peradeniya, Sri Lanka; 2 TACT, Department of Pharmacology, Sydney Medical School, University of Sydney, Sydney, Australia; 3 Department of Pharmacy, Faculty of Allied Health Science, University of Peradeniya, Peradeniya, Sri Lanka; 4 Department of Nephrology, Prince of Wales Hospital and Clinical School, University of New South Wales, Sydney, Australia; 5 Department of Medicine, Faculty of Medicine, University of Peradeniya, Peradeniya, Sri Lanka; 6 Department of Pathology, Faculty of Medicine, University of Peradeniya, Peradeniya, Sri Lanka; 7 Department of Biochemistry, Faculty of Medicine, University of Peradeniya, Peradeniya, Sri Lanka; 8 Clinical Toxicology Research Group, University of Newcastle, Newcastle, NSW, Australia; College of Health Sciences, Bayero University Kano, NIGERIA

## Abstract

**Background:**

Acute kidney injury (AKI) is a major complication of snake envenoming, but early diagnosis remains problematic. We aimed to investigate the time course of novel renal biomarkers in AKI following Russell’s viper (*Daboia russelii*) bites.

**Methodology/Principal findings:**

We recruited a cohort of patients with definite Russell’s viper envenoming and collected serial blood and urine samples on admission (<4h post-bite), 4-8h, 8-16h, 16-24h, 1 month and 3 months post-bite. AKI stage (1–3) was defined using the Acute Kidney Injury Network criteria. AKI stages (1–3) were defined by the Acute Kidney Injury Network (AKIN) criteria. There were 65 Russell’s viper envenomings and 49 developed AKI: 24 AKIN stage 1, 13 stage 2 and 12 stage 3. There was a significant correlation between venom concentrations and AKI stage (p = 0.007), and between AKI stage and six peak biomarker concentrations. Although most biomarker concentrations were elevated within 8h, no biomarker performed well in diagnosing AKI <4h post-bite. Three biomarkers were superior to serum creatinine (sCr) in predicting AKI (stage 2/3) 4-8h post-bite: serum cystatin C (sCysC) with an area under the receiver operating curve (AUC-ROC), 0.78 (95%CI:0.64–0.93), urine neutrophil gelatinase-associated lipocalin (uNGAL), 0.74 (95%CI:0.59–0.87) and urine clusterin (uClu), 0.81 (95%CI:0.69–0.93). No biomarker was better than sCr after 8h. Six other urine biomarkers urine albumin, urine beta2-microglobulin, urine kidney injury molecule-1, urine cystatin C, urine trefoil factor-3 and urine osteopontin either had minimal elevation, and/or minimal prediction for AKI stage 2/3 (AUC-ROC<0.7).

**Conclusions/Significance:**

AKI was common and sometimes severe following Russell’s viper bites. Three biomarkers uClu, uNGAL and sCysC, appeared to become abnormal in AKI earlier than sCr, and may be useful in early identification of envenoming.

## Author summary

Snake envenoming is a neglected tropical disease that causes death and severe effects in the rural tropics. Acute kidney injury is a major cause of morbidity in snake envenoming, but continues to be poorly recognised and early diagnosis is difficult. We aimed to investigate the time course of renal biomarkers in acute kidney injury, following Russell’s viper (*Daboia russelii*) envenoming. In a group of 65 definite Russell’s viper bites in Sri Lanka, acute kidney injury was common and sometimes severe. The severity of the acute kidney injury was associated with the blood venom concentrations. Three novel biomarkers, serum cystatin C, urine neutrophil gelatinase-associated lipocalin and urine clusterin became abnormal before serum creatinine, the current standard biomarker for kidney injury. These three novel biomarkers will be useful to diagnose snake envenoming and acute kidney injury. They provide insight into the time course of snakebite associated acute kidney injury.

## Introduction

Snake envenoming is a major medical problem in the rural tropics, particularly in South Asian farming communities where there are limited resources to prevent and treat snakebite [1]. Coagulopathy and neurotoxicity are well recognised major clinical syndromes of snake envenoming. Acute kidney injury (AKI) is a poorly recognised complication of snake envenoming and there is increasing evidence that snakebite associated AKI results in significant morbidity and mortality.[2,3] In severe cases it requires dialysis, which is rarely available in resource-poor regions. The pathophysiology of AKI in snake envenoming is poorly understood, and there is limited evidence of primary or direct nephrotoxins occurring in snake venoms. Several other mechanisms have been suggested, including AKI occurring secondary to hypotension, venom-induced consumption coagulopathy, rhabdomyolysis and microangiopathic haemolytic anaemia. [4–7] Bite to hospital time, hypotension, albuminuria, prolonged bleeding time, prolonged prothrombin time, low haemoglobin and high total bilirubin have been reported to be associated with AKI following snakebite.[8] Further study is required to better understand both the clinical features in these patients as well as the time course and severity of AKI.

Russell’s viper (*Daboia russelii*) is regarded as one of the most medically important venomous snakes in Asia. About 37,000 snakebite admissions are reported annually to Sri Lankan hospitals and 30 to 40% are estimated to be Russell’s viper bites, which lead to severe envenoming and fatalities.[1,9] Systemic envenoming from Russell’s viper bites most commonly results in venom-induced consumption coagulopathy and mild neurotoxicity. AKI appears to be less common, but severe AKI can result in a longer duration of hospital admission and risk of death.[10–12]

Currently, the diagnosis of AKI in snakebite relies on clinical signs such as oliguria or anuria in places where laboratory investigations are not available. In healthcare settings where laboratory investigations are available, either absolute or relative changes in serum creatinine (sCr) and blood urea nitrogen (BUN) are used for diagnosis. Similar to most forms of AKI, sCr and urea do not become abnormal early in the course of snakebite associated AKI and increase only after significant kidney injury has occurred, and when it may be too late for treatment such as antivenom. In addition, sCr is highly dependent on factors such as age, sex, race, muscle mass, nutritional status and volume of distribution.[13] Additional renal biomarkers need to be identified to improve the early diagnosis and treatment of snakebite associated AKI. Extensive investigation has led to the identification and evaluation of many urine and serum proteins that are potential renal biomarkers and directly measure kidney injury rather than function. [14] There are a number of cellular proteins released into urine that have been used to monitor kidney injury in animals and humans. These include kidney injury molecule-1 (uKIM-1), albumin (uAlb), total protein, beta2-microglobulin (uβ2M), cystatin C (uCysC), clusterin (uClu), trefoil factor-3 (uTFF3), N-acetyl-β-D-glucosaminidase (uNAG), neutrophil gelatinase-associated lipocalin (uNGAL), osteopontin (uOPN) and interleukin-18 (IL-18).[15–17] Recent studies of AKI in poisoned patients have demonstrated the usefulness of these biomarkers, including in glyphosate [18–19] and paraquat poisoning, [20,21] in animals and humans.

This study aimed to investigate a range of novel and established renal biomarkers in Russell’s viper envenoming to determine if they had greater diagnostic accuracy compared to the conventional tests in identifying snakebite associated AKI and to establish the time course of the changes of these renal biomarkers in snakebite associated AKI.

## Materials and methods

This was a prospective cohort study to investigate the diagnostic utility of a range of serum and urine renal biomarkers in systemic envenoming following definite Russell’s viper bites.

The study had approval from the Ethical Review Committee, Faculty of Medicine, University of Peradeniya, Sri Lanka, the University of New South Wales and University of Newcastle, Australia. All patients gave informed written consent for the collection of blood samples, urine samples and clinical information. All participants in the study were adults.

All adult patients with suspected or confirmed Russell’s viper bites (symptomatic patients with Russell’s viper bite and local or systemic envenoming, neurological or haematological features) presenting to the General Hospital, Polonnaruwa, from April 2012 to March 2015 were approached for consent. Patients <16 years, pregnant patients or patients with definite bites by snakes other than a Russell’s viper were excluded. Only patients with confirmed Russell’s viper envenoming were included in the study based on the detection of Russell’s viper venom antigens in serum using venom specific enzyme immunoassay.[22,23] In addition, patients were only eligible if they had two or more serum samples available, one or more urine samples and at least one follow up serum sample available.

The following information was collected from all patients prospectively: demographics (age and sex), medication history, time of the bite, clinical effects (local effects, clinical features of coagulopathy, bleeding and neurotoxicity), complications and antivenom treatment (dose and time of administration). All demographic and clinical data were collected on a pre-formatted data sheet.

Blood samples (8 to 10 ml) were collected from all patients in tubes without anticoagulants on admission and within the first 4h of the bite, between 4 and 8h, between 8 and 16h and between 16 and 24h, post-bite. Urine samples were collected in parallel with blood collection and then daily until discharge. After discharge, all patients were followed-up at one month and three months at the clinic or their home. At the follow up clinical data as well as serum and urine samples were collected for biomarker studies. All serum and urine samples were centrifuged, and then aliquoted and frozen initially at -20°C, and then transferred to -80°C within 2 weeks of collection.

Serum samples from all recruited patients were measured for snake venom using a sandwich enzyme immunoassay as previously described.[22,23] Rabbit IgG was raised against Sri Lankan Russell’s viper (*D*. *russelii*). Antibodies were then bound to microplates and separately conjugated to biotin for the sandwich enzyme immunoassay, detecting with streptavidin-horseradish peroxidase. The lower limit of detection for the assay was 2ng/ml. Absorbance was measured in triplicate in all samples and the average absorbance then converted to a venom concentration with a standard curve of serial venom dilutions. The coefficient of variation for triplicate wells was <10% for low and high absorbance. In cases in which pre-antivenom samples were not available or venom could not be detected in pre-antivenom samples, the post-antivenom samples underwent dissociation treatment (HCl-glycine), which dissociates venom components from antivenom molecules.[24] These post-antivenom samples then had Russell’s viper venom concentrations measured with the same sandwich enzyme immunoassay.

Biomarker assays were done batchwise on all samples. Serum creatinine and urine creatinine (uCr) concentrations were measured by the modified Jaffe method (kinetic Jaffe reaction). Serum Cystatin C (sCysC) was also quantified in the same serum sample using a microparticle-enhanced immune turbidimetric method on a clinical chemistry analyser (Konelab ThermoFisher, Waltham, MA), according to manufacturer’s recommendations.

Duo Set enzyme-linked immunosorbent assay (ELISA) kits (R&D systems) were used to quantify uKIM-1 and uClu. The sandwich ELISA technique was undertaken according to the manufacturer's directions. Microtitre plates were coated with the capture antibody and incubated overnight at room temperature (25°C) in a microplate incubator/shaker (Stat Fax 2200). Serially diluted standards, quality controls, dilution buffer (= blank) and samples, were then loaded in duplicate and incubated at room temperature. Plates were washed and then loaded with conjugate solution, incubated for the recommended time duration and then washed. The substrate solution was then added to each well in the next step and incubated. Finally, the colour reaction was stopped by adding stop solution. The absorbance of each well was measured with a microplate reader at a wavelength of 450nm.

The other urinary biomarkers uAlb, uβ2M, uCysC, uNGAL, uOPN and uTFF3 were quantified using BIO-Plex ProRBM Human Kidney Toxicity Assays panel 2 on the Bio-Plex 200 system (BIO-RAD, USA). Biomarker concentrations are reported as the absolute concentration.

The acute kidney injury network (AKIN) criteria were used to define AKI based on sCr, which is the commonly used definition in clinical studies assessing renal biomarker profiles.[18,20] In brief, AKIN stage 1 [mild] was defined as an absolute increase in sCr of more than or equal to 0.3 mg/dl (≥ 26.4 μmol/l) or a relative increase by more than or equal to 150% to 200% (1.5 to 2 fold) from baseline. AKIN stage 2 [moderate] was defined as a relative increase in sCr of >200% to 300% (>2 to 3 fold) from baseline and AKIN stage 3 [severe] as a relative increase in sCr of >300% (>3 fold) from baseline (or an absolute increase of sCr by more than or equal to 4.0 mg/dl [≥354 μmol/l] with an acute increase of at least 0.5 mg/dl [44 μmol/l]). The baseline sCr was defined as the lowest sCr concentration greater than 0.4 mg/dl measured on follow up or on the admission if it was lower than at one or three month follow. For the majority of the analyses, patients with AKIN stage 2 and AKIN stage 3 were grouped together because of the smaller numbers and the similarity in these groups (Table 1).

**Table 1:** Patient demographic and clinical data.

	No AKI	AKIN1	AKIN2	AKIN3
Number	16	24	13	12
Males	10 (63%)	21(88%)	11(85%)	9(75%)
Median age (years) (range)	30 (15–50)	41 (16–66)	51 (18–71)	49.5 (22–76)
Median weight (kg) (range)	54 (40–66)	55 (46–68)	65 (45–70)	56.5 (40–70)
Median time to admission	1h 27min	1h 30min	1h 10min	1h 50min
Site of bite				
Lower Limb	14	22	12	12
Upper Limb	2	1	-	-
Clinical manifestations				
Local signs and symptoms (pain and/or swelling)	14 (88%)	18 (75%)	12 (92%)	12 (100%)
Positive WBCT20	8 (53%)	19 (86%0	8 (67%)	12 (100%)
Bleeding (Haematemesis/ haematuria/ bleeding from bite site/ IV site)	2 (13%)	5 (21%)	6 (46%)	4 (33%)
Neurotoxicity	9 (56%)	10 (42%)	9 (69%)	8 (67%)
Median venom concentration (ng/ml) (range)	59 (2–1456) (n = 10)	50 (2–972) (n = 16)	351 (2–3125) (n = 13)	483 (2–2244) (n = 10)
Treatment with antivenom	15 (94%)	22 (92%)	12 (92%)	12 (100%)

Neurotoxicity was the presence of any of ptosis, diplopia, blurred vision

Continuous data were reported as medians, interquartile ranges (IQR) and ranges, and categorical data were summarized as proportions with 95% confidence intervals (CI). The time courses of all biomarker concentrations were compared between patients who developed AKI (mild, moderate or severe) and those who did not. Comparison and correlation between the ordered groups (No AKI, AKI stage 1, AKI stage 2 and AKI stage 3) for venom concentrations and biomarkers were undertaken using Kendall’s tau test in SPSS with a one-tailed test (only testing for smallest to largest). Due to the limited numbers and the importance in identifying more severe AKI, further statistical analysis was done grouping moderate and severe AKI patients together (AKIN stages 2 and 3), and comparing these with those who did not develop AKI, and those who developed mild AKI (AKIN Stage 1)–this was the primary diagnostic analysis. The diagnostic performance of each biomarker for snakebite associated AKI within each time interval was assessed by area under the receiver operator characteristic curve (AUC–ROC) and compared with the ‘No AKI’ patients. Correlations between biomarkers were assessed using peak biomarker concentrations within 24h of Russell’s viper bite. Statistical analysis was performed using GraphPad Prism software version 7.03 (San Diego, USA), except for SPSS Statistics (IBM 2017).

## Results

**Fig 1:**
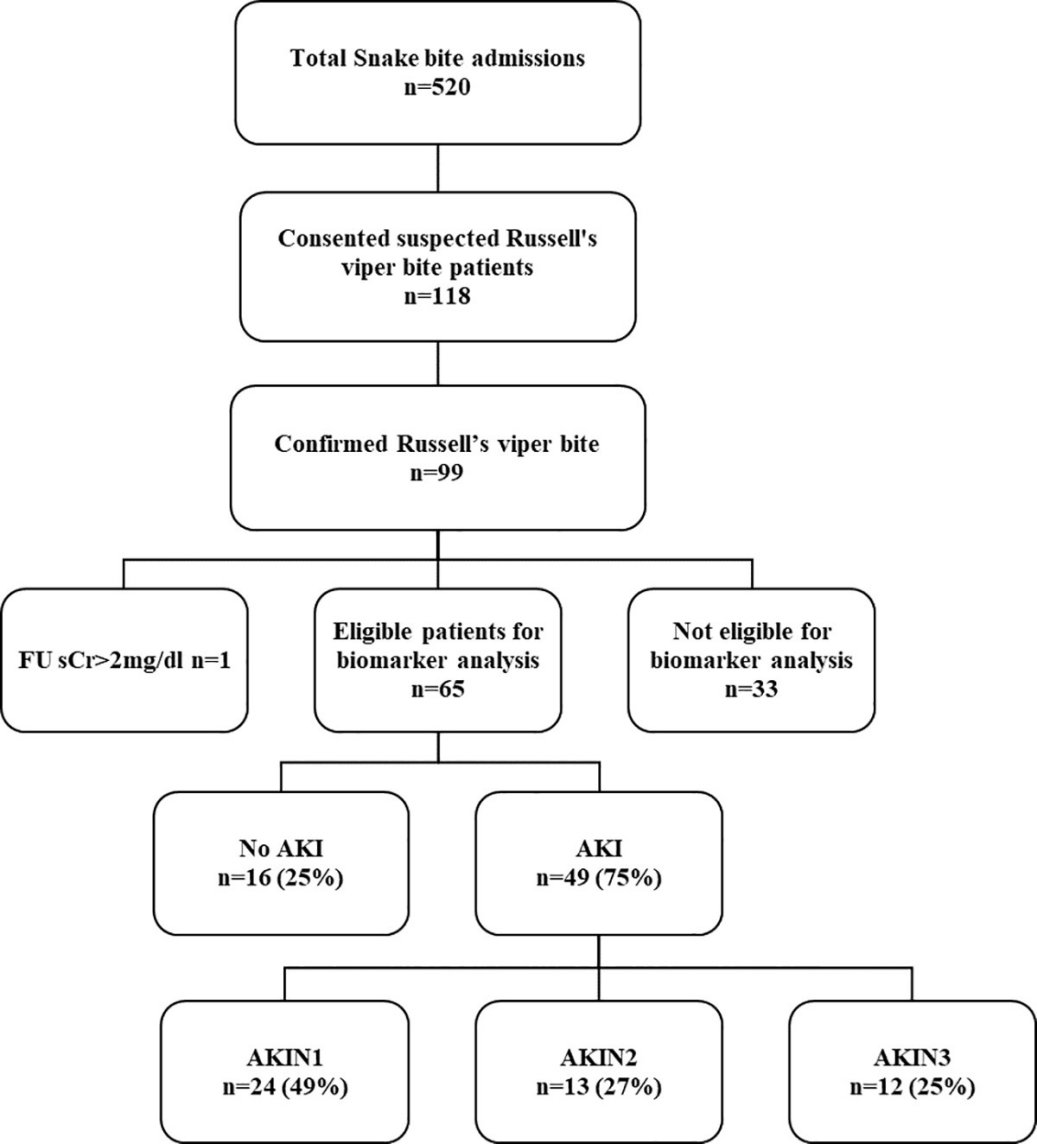
Flow chart of patient inclusion and acute kidney injury grading. Thirty three patients who did not have two or more serum samples, one or more urine samples and at least one follow up (FU) serum sample available were excluded. One patient who had a serum creatinine value >2 mg/dl even at the follow up (FU) was excluded from the study as it is above the level of normal healthy adult.

There were 520 patients admitted with snakebites during the study period, and 118 of these were suspected Russell’s viper bites. From this, 99 patients were positive for Russell’s viper venom and 65 patients with sufficient serum and urine samples were included in the analysis (Fig 1). One patient consistently had a sCr value >2mg/dl throughout the hospital stay and also had a follow-up sCr>2mg/dl. This patient was excluded assuming that he had chronic kidney disease with no acute kidney injury (S1 Fig). Patient demographic and clinical data are summarized for patients by AKIN stages (Table 1). Fifty-one patients were males (79%) and the median age was 42 y (IQR: 31 to 50 y; Range: 16 to 76 y). The median time from bite to admission was 1h 27min (IQR: 46 min to 2h 5min; Range: 10min to 26h).

**Fig 2:**
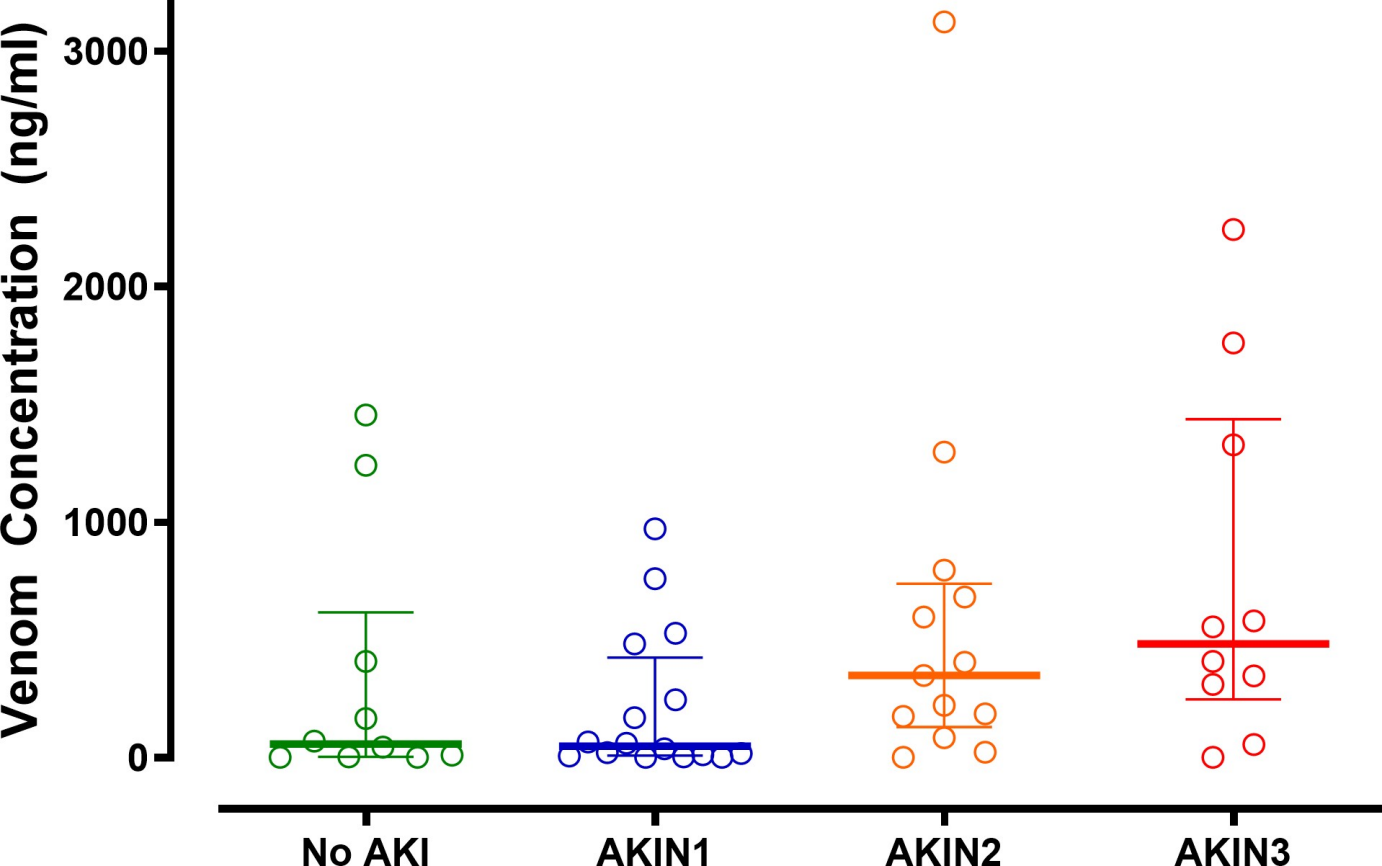
Scatter plots of the pre-antivenom Russell’s viper venom concentrations (median and interquartile range) for each of the acute injury groups.

Of the 65 eligible patients, 49 developed AKI (75%); 24/65 (37%) developed AKIN stage 1 (mild), 13/65 (20%) developed AKIN stage 2 (moderate) and 12/65 (18%) developed AKIN stage 3 (severe). Patients with stage 2 or 3 AKI (moderate or severe) were older and more likely to have other features of envenoming, including coagulopathy and neurotoxicity. Among 49 patients with pre-antivenom concentrations available, there was a significant increasing correlation between median venom concentrations for patients with no AKI (58 ng/mL), AKIN stage 1 (50 ng/mL), AKIN stage 2 (351 ng/mL) and AKIN stage 3 (483 ng/mL; Fig 2 p = 0.007 Kendall’s tau test; correlation coefficient 0.271). Antivenom was administered to 61 patients. The decision to administer antivenom in the hospital was based on a positive 20-minute whole blood clotting test (WBCT20) in 47 patients, development of non-specific clinical features of systemic envenoming in five and neurological features in nine patients.

**Fig 3:**
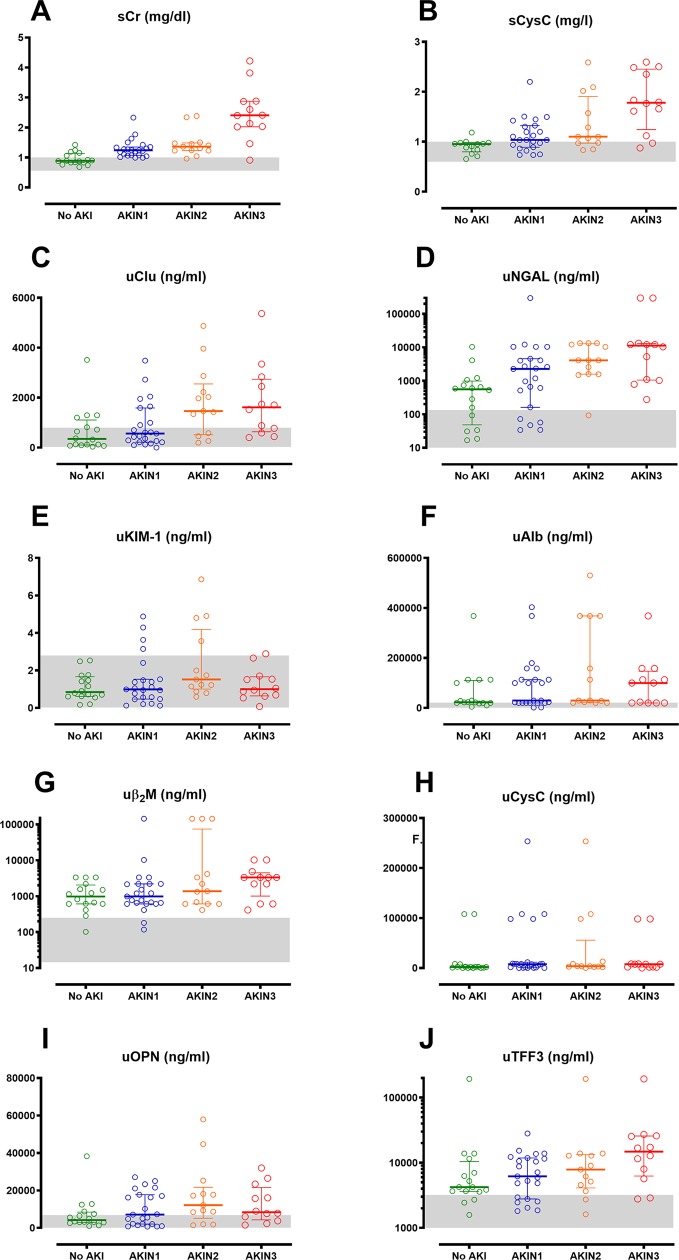
Maximum renal biomarker concentrations within 24 hours of bite. Scatter plots of the maximum biomarker concentrations reached within 24 h post-bite for patients with no acute kidney injury (No AKI; green), mild AKI (grade 1; blue), moderate AKI (grade 2; orange) and severe AKI (grade 3; red), for serum creatinine (sCr; Panel A), serum cystatin C (sCysC; Panel B), urinary clusterin (uClu; Panel C), urinary neutrophil gelatinase-associated lipocalin (uNGAL; Panel D), kidney injury molecule-1 (uKIM-1; Panel E),albumin (uAlb; Panel F),beta2-microglobulin (uβ2M; Panel G), urinary cystatin C (uCysC; Panel H), osteopontin(uOPN; Panel I) and trefoil factor-3 (uTFF3; Panel J).

The maximum renal biomarker concentration reached within 24 h post-bite for each patient group, based on the AKIN severity, are illustrated in Fig 3. All biomarkers peaked within 24 h post-bite (S2 Fig). There was a significant correlation between different AKI groups for most of the peak biomarker concentrations, increasing from no AKI to severe AKI (Kendall’s tau test; S1 Table). This was most significant with the greatest increase from no AKI to severe AKI for sCr, then sCysC, uClu and uNGAL (p<0.01; correlation coefficient 0.339 to 0.593). The remaining three biomarkers were not significantly different between the four groups.

**Fig 4:**
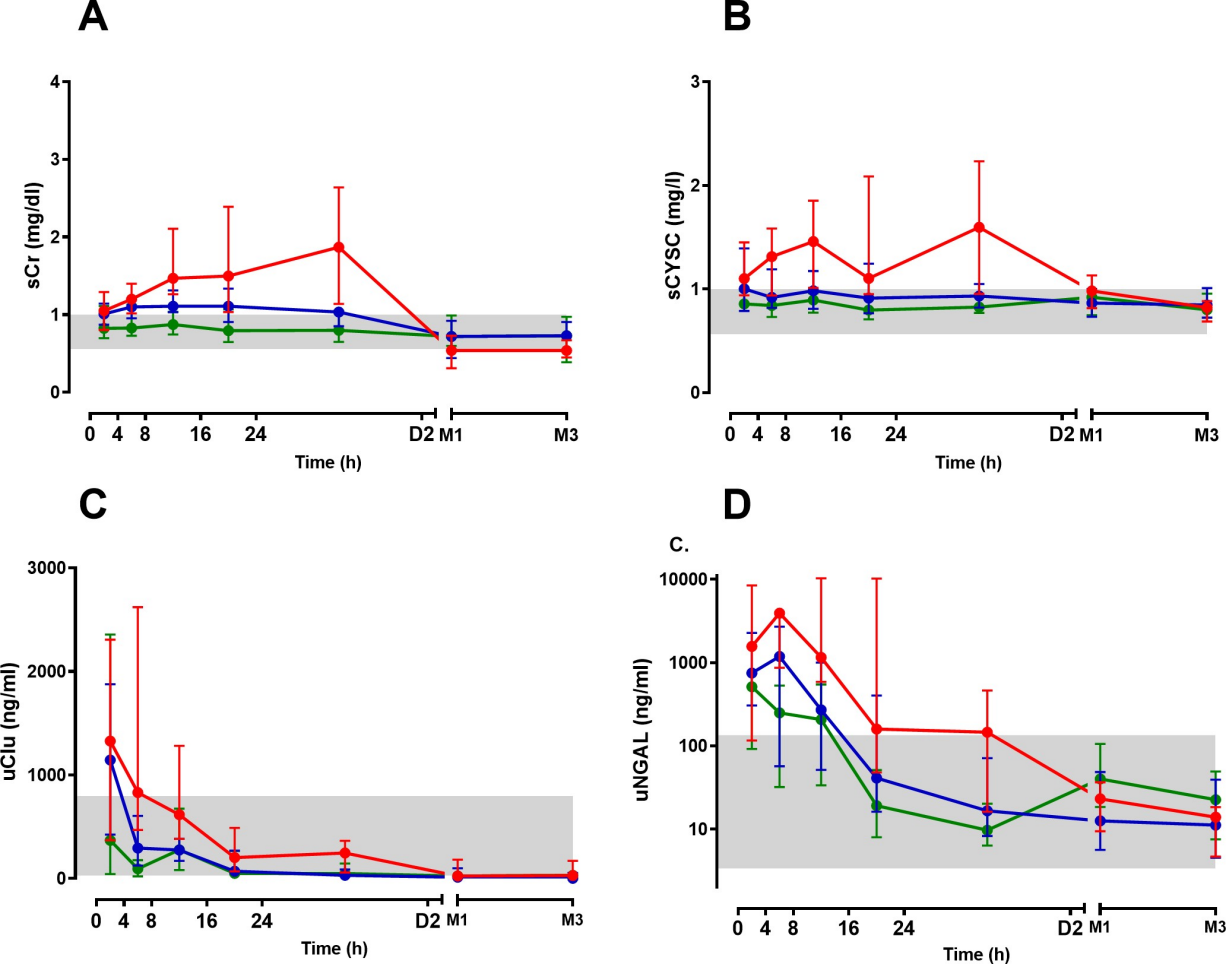
Time course of the median biomarker concentrations (with interquartile ranges) for each of the three patient groups (No acute kidney injury [No AKI; green], mild AKI [blue] and moderate to severe AKI [red]). AKI, AKIN stage 1 and AKIN stage 2/3) post-bite for 2 days, then at 1 and 3 months, including serum creatinine (sCr; Panel A), serum cystatin C (sCysC; Panel B), urinary clusterin (uClu; Panel C) and urinary neutrophil gelatinase-associated lipocalin (uNGAL; Panel D). The grey shaded area is the normal range based on respective biomarkers measured in healthy individuals.

Serum creatinine increased over the first 8 to 16 h in patients with moderate to severe AKI (AKIN stage 2/3), with a median peak sCr occurring between 8 to 16h post-bite (Fig 4 and S3 Fig). In mild AKI (AKIN stage 1) there was less of an increase between 8 to 16h post-bite, with an increase of 50% by 8 to 16h post-bite. sCysC rapidly increased in patients with moderate/severe AKI with median peak sCysC occurring between 8 to 16h post-bite. There was not a significant increase in sCysC in patients with mild AKI (Fig 4 and S3 Fig).

The urinary biomarker uNGAL was abnormal in all cases of AKI on admission, then further increased and peaked between 4 to 8h post-bite (Fig 4 and S3 Fig). It then decreased into the normal range between 16 to 24h post-bite. The biomarker uClu was abnormal and peaked within the first 4h of the bite in all cases of AKI, compared to patients without AKI. At 4 to 8 h, uClu decreased into the normal range for mild AKI, but remained higher and abnormal for moderate/severe AKI. It then decreased to within the normal range for moderate/severe AKI between 8 to 16h post-bite.

The other six renal biomarkers provided less diagnostic information, with patients with AKI having biomarker concentrations in the normal reference range or there being no separation in biomarker concentrations between no AKI cases and AKI cases. uKIM-1 and uOPN fluctuated within the normal reference range. uCysC and uβ2M were above the normal range on admission for all cases of AKI, including no AKI, and then decreased over time. uAlb was abnormal in moderate/severe AKI between 16 to 24 h, but not before or after this. uTFF3 increased 4 to 8 h post-bite for moderate/severe AKI, but not mild AKI, then decreased at 8 to 16 h. (S2 Fig and S3 Fig).

**Fig 5:**
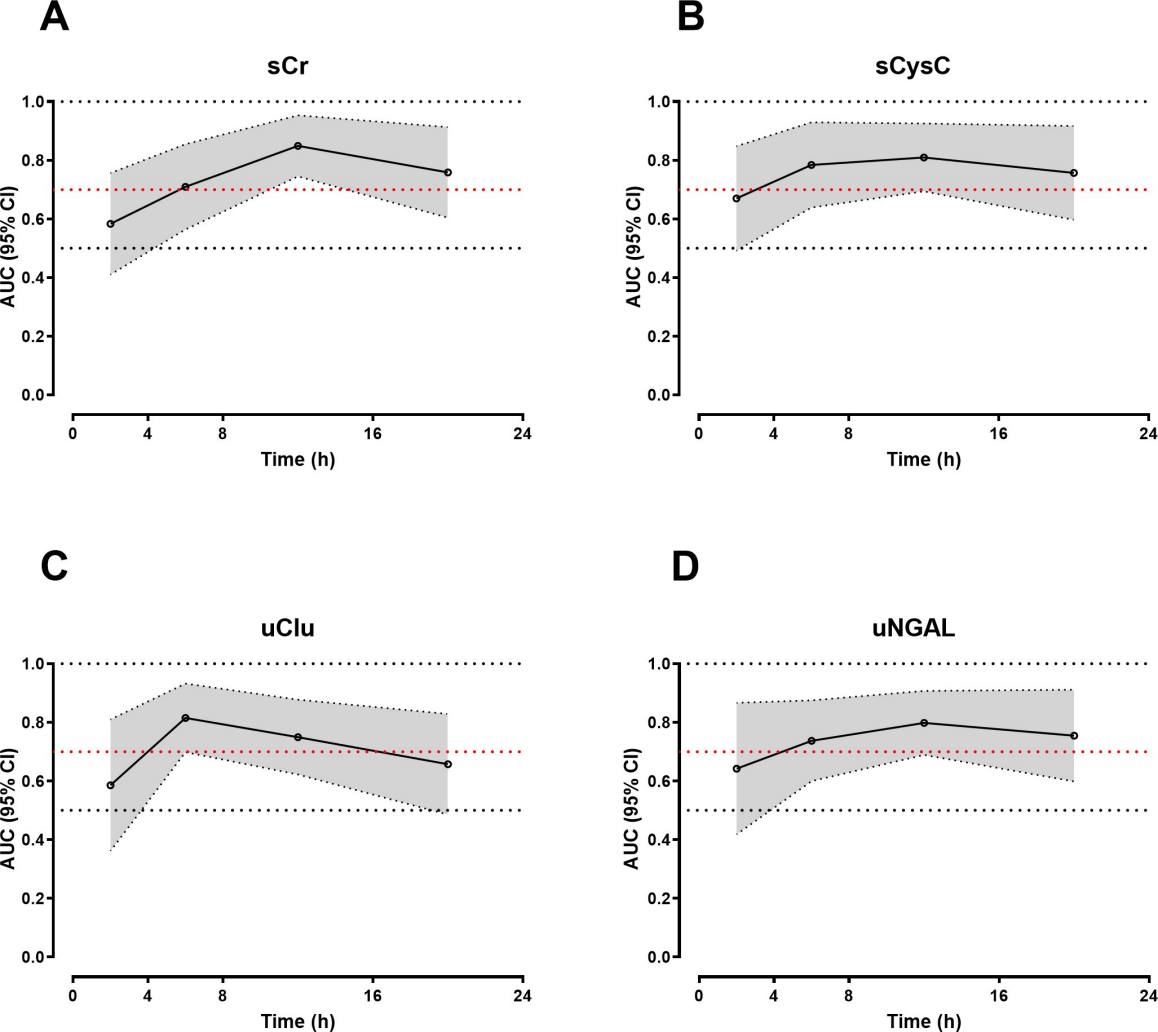
Plots of the AUC-ROCs versus time for the best functional and structural biomarkers in detecting moderate/severe AKI versus No AKI/mild AKI, includingserum creatinine (sCr; Panel A), serum cystatin C (sCysC; Panel B), urinary clusterin (uClu; Panel C) and urinary neutrophil gelatinase-associated lipocalin (uNGAL; Panel D).The dark black line represents the AUC-ROCs and the shaded area covers the 95% confidence intervals (CI) for the AUC-ROC.

AUC-ROC curves demonstrated that the peak sCr in the first 24 h was the best predictor of moderate to severe AKI versus mild AKI/No AKI, with an AUC-ROC of 0.84 (95% CI: 0.73–0.94). This compared to an AUC-ROC of 0.76 (95% CI: 0.63–0.89), 0.78 (95% CI: 0.67–0.89) and 0.76 (0.64–0.88) for sCysC, uNGAL and uClu respectively (S4 Fig). These three biomarkers performed better than sCr at 4 to 8 h post-bite in predicting moderate/severe AKI (Table 2 and Fig 5). However, no biomarker performed better than sCr more than 8 h post-bite.

**Table 2:** The area under the curve of the receiver operator characteristic (AUC-ROC) curve (with 95% confidence intervals) for biomarker concentrations predicting moderate to severe acute kidney injury (AKI) versus none or mild AKI, in each of the four time periods within the first 24h of the bite.

	AUC-ROC (95% CI)			
	0-4h	4-8h	8-16h	16-24h
**sCr**	0.58 (0.41–0.76)	0.71 (0.56–0.85)	0.85 (0.74–0.95)	0.76 (0.60–0.91)
**sCysC**	0.67 (0.49–0.85)	0.78 (0.64–0.93)	0.80 (0.69–0.92)	0.76 (0.59–0.92)
**uNGAL**	0.64 (0.42–0.87)	0.74 (0.59–0.87)	0.79 (0.68–0.90)	0.75 (0.59–0.91)
**uClu**	0.58 (0.36–0.81)	0.81 (0.69–0.93)	0.75 (0.62–0.87)	0.66 (0.48–0.83)
**uCysC**	0.52 (0.29–0.74)	0.66 (0.52–0.81)	0.69 (0.56–0.83)	0.64 (0.47–0.81)
**uTFF3**	0.56 (0.35–0.77)	0.66 (0.51–0.81)	0.67 (0.53–0.82)	0.65 (0.47–0.82)
**uKIM-1**	0.59 (0.37–0.82)	0.60 (0.45–0.76)	0.61 (0.46–0.76)	0.59 (0.41–0.78)
**uβ2M**	0.63 (0.43–0.83)	0.67 (0.52–0.81)	0.56 (0.40–0.71)	0.66 (0.48–0.83)
**uOPN**	0.62 (0.41–0.83)	0.56 (0.41–0.72)	0.65 (0.51–0.79)	0.66 (0.49–0.84)
**uAlb**	0.59 (0.39–0.81)	0.56 (0.40–0.71)	0.62 (0.47–0.76)	0.70 (0.53–0.87)

Neither sCr, nor any new biomarker performed well in predicting moderate/severe AKI within 4 h of the bite (Table 2), although uClu appeared to distinguish all cases of AKI versus no AKI in the first 4 h (Fig 4). In the period 4-8h post-bite, the three biomarkers sCysC, uNGAL and uClu appeared to perform better than sCr in predicting moderate/severe AKI versus mild AKI/NoAKI, with AUC-ROC of 0.78 (95% CI: 0.64–0.93), 0.74 (95% CI: 0.59–0.87) and 0.81 (95% CI: 0.69–0.93) respectively (Table 2, Fig 5).

Urinary KIM-1, uAlb, uCysC, uβ2M, uOPN and uTFF3 all performed poorly, with the AUC-ROCs < 0.7 for all time points 24 h post-bite (Table 2 and S5 Fig).

## Discussion

Acute kidney injury is usually defined based on changes in sCr, both in clinical practice and in research. We observed increased urinary renal biomarkers including uClu, uNGAL, uAlb, uβ2M, uCysC, and uTFF3 in patients with Russell’s viper envenoming. Many of these showed minimal difference between those who did and did not then develop moderate/severe AKI. uClu, uNGAL and uCysC predicted moderate/severe AKI better than sCr in a small window from 4 to 8 h post-bite. However, uClu was abnormal for all stages of AKI versus no AKI in the first 4 h post-bite, which was better than sCr. uNGAL was abnormal in all patients within 4 h, so was unable to predict AKI in this period but may potentially identify envenomed patients. The other six urinary biomarkers performed poorly at any time after the bite compared to sCr. To some extent this confirms the limited sensitivity of creatinine in detecting minor degrees of kidney injury.

The three novel biomarkers uClu, uNGAL and sCysC appeared to be better diagnostic biomarkers of AKIN 2/3 compared to sCr from 4 to 8 h post-bite and might allow earlier interventions for snakebite patients who are developing AKI. Closer inspection of Fig 4 suggest some differences worth noting in the first 4 h period post-bite. In the first 4 h uClu was much higher in all cases of AKI (AKIN 1 and AKIN 2/3) versus no AKI (Fig 4), so may provide the earliest indicator of AKI. However, it did not predict patients who developed more severe AKI (AKIN 2/3), compared to those with mild (sub-clinical) AKI, which our primary analysis investigated (Table 2). In contrast, uNGAL was abnormal in all patients on admission (within 4 h) and appeared to show the best graded response, with separation of all three groups–no AKI, AKIN 1 and AKIN 2/3)–for all time periods. Again, this was not reflected in our primary analysis comparing AKIN 2/3 to No AKI or AKIN 1.

uNGAL became rapidly abnormal in nearly all envenomed patients in our series (Figs 3 and 4). This suggests it may in fact be more beneficial in the early identification of envenoming, rather than snakebite associated AKI. However, for this to be an effective diagnostic assay, it needs to detect envenoming within 4 h of the bite so that antivenom can be administered as early as possible. Further research is required to investigate the value of NGAL in the detection of envenoming within 4 h of the bite, which requires having a non-envenomed comparison group. sCr, sCysC and uClus were all normal in the No AKI group, including for the first 8 h, so would not be useful as early diagnostic tests for ‘envenoming’.

NGAL expression is up-regulated within the first few hours after ischaemic renal injury in animal models and has been identified as an early biomarker for diagnosing AKI.[25] Urinary and serum NGAL represent sensitive, specific, and highly predictive early biomarkers of AKI following common clinical settings in which AKI occurs, including the intensive care unit [26], following cardiac surgery, after contrast administration for percutaneous coronary interventions, in contrast-induced nephropathy in children, and following kidney transplantation in adults. [27–30]. Previous studies have suggested that plasma NGAL may be a useful biomarker of AKI in snakebite [31,32]. Plasma NGAL within 48 h of the bite predicted AKI based on RIFLE criteria. [31] In that study there was no confirmed snake identification, and the early samples were collected within 10 h to evaluate the early diagnostic utility of NGAL, but too late for early antivenom administration [31]. Another study reported plasma NGAL within 8 h was a good predictor of “AKI”. However, they used an arbitrary definition for AKI (sCr > 1.4 mg/dL at 24 h). There was also little data provided on the time to admission, types of snake bite or confirmation of snake bite. It is not possible to determine when in the 8 h post-bite period measurements were made.[32] Neither study compared sNGAL to creatinine or other biomarkers at the same time points. The poor definition of the time of NGAL measurement and the absence of definite snakebite confirmation, make it difficult to compare our results with these two studies [31,32]. Few recent clinical and [18,20,33] pre-clinical [34] studies investigated urinary NGAL in poisoning and showed a modest diagnostic performance (AUC-ROC = 0.7 to 0.8) in toxicant-induced AKI within 24 h of poisoning [18,20,33,34]

Clusterin increases in the kidney of rats after several causes of AKI, such as ischemia/reperfusion injury, toxicant-induced kidney injury and unilateral urethral obstructions.[35,36] Clusterin expression occurs in both proximal and distal tubule cells. No previous studies have explored the role of urinary clusterin as a diagnostic marker of AKI following snake envenoming. However, clusterin performed modestly well in predicting AKI following paraquat poisoning [20] and very well in predicting AKI (as delayed graft function) 4, 8 and 12 hours after kidney transplantation [30].

A number of immunoassays are now in use for the measurement of NGAL in urine and blood. Currently, point-of-care devices and platforms (using immunoassay methods) that provide results in less than 1 hour are available in the clinical setting. [37] A point-of-care assay is also available for the measurement of sCysC (Eurolyseron the smart or CUBE laboratory photometer), based on a latex enhanced immunoturbidimetric assay method. Point of care devices for measuring clusterin in blood or urine are currently not available. The utility of available point of care devices and platforms needs to be explored in larger populations, particularly to determine if they can predict patients developing AKI in 3 to 6 h so that it might inform the timely use of specific interventions for AKI.

Although this is the first comprehensive study to evaluate the diagnostic utility of several structural and functional markers of AKI following snake envenoming, there are a number of limitations. The most important limitation of our study is that AKI was defined by the acute kidney injury network criteria, which relies on the rise in serum creatinine, and not by the gold standard–histopathology–which is invasive and not a routine investigation.

A small number of patients were excluded because of low or high creatinine values, or unknown timing of the bite. All creatinine values <0.4 mg/dl were excluded because it was assumed that they were due to collection or analytical errors. There was one patient who was categorized in AKIN 1 stage, with an unknown bite time and could not be included in the analysis of the time course of the biomarkers. One patient had consistently high sCr values >2mg/dl and was excluded because it was assumed that he had chronic kidney disease (S1 Fig).

Ideally the baseline sCr should be taken prior to the onset of AKI.[38] However, as these were not available for our patients we used the lowest value of sCr measured (generally at follow-up or on admission) as in most previous studies.[18,38]

Another limitation of the study was that most patients received antivenom and a few received other medications for antivenom reactions or local infection. Antivenom administration is not reported to cause renal injury, except for delay hypersensitivity reactions–serum sickness–which will not cause any early renal involvement. Antihistamines, corticosteroids and antibiotics were administered in a few patients, but are rare causes of acute kidney injury and were administered hours after the bite or longer.

AKI was common and associated with higher venom concentrations; there was increased risk for older patients. The urinary biomarker uNGAL rose rapidly within 4 h for all patients with Russell’s viper envenoming, and uClu was abnormal within 4 h in all patients with all severities of AKI. These two biomarkers in addition to sCysC predicted more severe kidney injury better than sCr in the period 4 to 8 h post-bite. Further investigation will determine the role of NGAL in the diagnosis of envenoming per se, and uClu and sCysC in early prediction of snakebite associated AKI.

## Supporting information

S1 Table Peak biomarker concentrations reached within 24h post-bite. Absolute biomarker concentrations are presented as median (+/-IQR) (No AKI, AKIN stage 1, AKIN stage 2 and AKIN stage 3 were compared and correlated using Kendall’s tau test with one-tailed p-value)
(PDF)

S1 Fig Time course of the serum creatinine concentration in the one patient with chronic kidney disease. The grey shaded area is the normal range based on serum creatinine measured in healthy individuals.
(TIF)

S2 Fig Time course of the median biomarker concentrations *(± IQR*) following Russell’s viper bite for 2 days then at 1 and 3 months, including urinary kidney injury molecule-1 (uKIM-1; Panel A), urinary albumin (uAlb; Panel B), urinary beta2-microglobulin (uβ2M; Panel C), urinary cystatin C (uCysC; Panel D), urinary osteopontin (uOPN; Panel E) and urinary trefoil factor-3 (uTFF3; Panel F). Patients without acute kidney injury (NO AKI; green), mild AKI (blue), moderate to severe AKI (red). The grey shaded area is the normal range based on respective biomarkers measured in healthy individuals.
(TIF)

S3 Fig Absolute changes in each of the biomarkers following Russell’s viper bite for each individual patients over the first 2 days: [serum creatinine (sCr; Panel A), serum cystatin C (sCysC; Panel B), urinary clusterin (uClu; Panel C), urinary neutrophil gelatinase-associated lipocalin (uNGAL; Panel D), urinary kidney injury molecule-1 (uKIM-1; Panel E), urinary albumin (uAlb; Panel F), urinary beta2-microglobulin (uβ2M; Panel G),urinary cystatin C (uCysC; Panel H), urinary osteopontin (uOPN; Panel I) and urinary trefoil factor-3 (uTFF3; Panel J)]. Patients without acute kidney injury (No AKI; green), mild AKI (blue), moderate to severe AKI (red). The grey shaded area is the normal range based on respective biomarkers measured in healthy individuals.
(TIF)

S4 Fig Receiver operator characteristic (ROC) curve analysis of the peak in each biomarker concentration within 24 hours post-bite detecting moderate to severe AKI versus No AKI/ mild AKI, for serum creatinine (sCr; red line), serum cystatin C (sCysC; pink line), urinary clusterin (uClu; blue line) and urinary neutrophil gelatinase-associated lipocalin (uNGAL; green line).
(TIF)

S5 Fig Plots of the AUC-ROCs versus time for six renal biomarkers in detecting moderate/severe AKI versus No AKI/ mild AKI, including urinary kidney injury molecule-1 (uKIM-1; Panel A),urinary albumin (uAlb; Panel B), urinary beta2-microglobulin (uβ2M; Panel C),urinary cystatin C (uCysC; Panel D), urinary osteopontin (uOPN; Panel E),and urinary trefoil factor-3 (uTFF3; Panel F). The dark black line represents the AUC-ROCs and the shaded area covers the 95% confidence intervals (CI) for the AUC-ROC.
(TIF)
