# Supplementary material for: Early identification of acute kidney injury in Russell’s viper (Daboia russelii) envenoming using renal biomarkers
Source: PLoS Negl Trop Dis. 2019 Jul 1;13(7):e0007486. doi: 10.1371/journal.pntd.0007486 (PMC6625728; doi:10.1371/journal.pntd.0007486)
Supplement: S1 Table — Absolute biomarker concentrations are presented as median (+/-IQR) (No AKI, AKIN stage 1, AKIN stage 2 and AKIN stage 3 were compared and correlated using Kendall’s tau test with one-tailed p-value) (PDF) [file pntd.0007486.s001.pdf]

S1 Table

Biomarker	No AKI (n=16)	AKIN1 (n=24)	AKIN2 (n=13)	AKIN3 (n=12)	p value	Correlation coefficient
sCr (mg/dl)	0.89 (0.83-1.1)	1.2 (1.1-1.4)	1.4 (1.2-1.5)	2.4 (2-2.9)	<0.001	0.593
sCysC (mg/l)	0.95 (0.80-0.98)	1.0 (0.89-1.3)	1.1 (0.97-1.9)	1.8 (1.2-2.5)	<0.001	0.434
uKIM1 (ng/ml)	0.84 (0.61-1.7)	0.99 (0.47-1.5)	1.5 (1-4.2)	1.1 (0.64-1.7)	0.12	0.112
uClu (ng/ml)	347 (101-1103)	563 (218-1588)	1463 (517-2549)	1614 (633-2738)	0.0002	0.339
uAlb (ng/ml)	22629 (19845-109526)	29279 (19785-112856)	29279 (21578-368156)	99539 (20024-146823)	0.061	0.152
uβ2M (ng/ml)	983 (611-2050)	989 (611-2212)	1385 (611-74565)	3350 (1011-4478)	0.016	0.209
uCysC (ng/ml)	2024 (160-6328)	7594 (925-11678)	3667 (2301-55705)	7594 (1773-8182)	0.66	0.145
uNGAL(ng/ml)	558 (48-985)	2266 (162-4564)	4097 (1583-12694)	11229 (1048-12969)	<0.001	0.418
uOPN (ng/ml)	4178 (2972-8427)	7113 (2115-17705)	12189 (5164-21691)	8379 (4319-21576)	0.028	0.181
uTFF3 (ng/ml)	4228 (3610-10427)	6198 (2779-11856)	7875 (4116-13382)	14837 (6269-25756)	0.006	0.238
