# Supplementary figures and images for: Early identification of acute kidney injury in Russell’s viper (Daboia russelii) envenoming using renal biomarkers
Source: PLoS Negl Trop Dis. 2019 Jul 1;13(7):e0007486. doi: 10.1371/journal.pntd.0007486 (PMC6625728; doi:10.1371/journal.pntd.0007486)

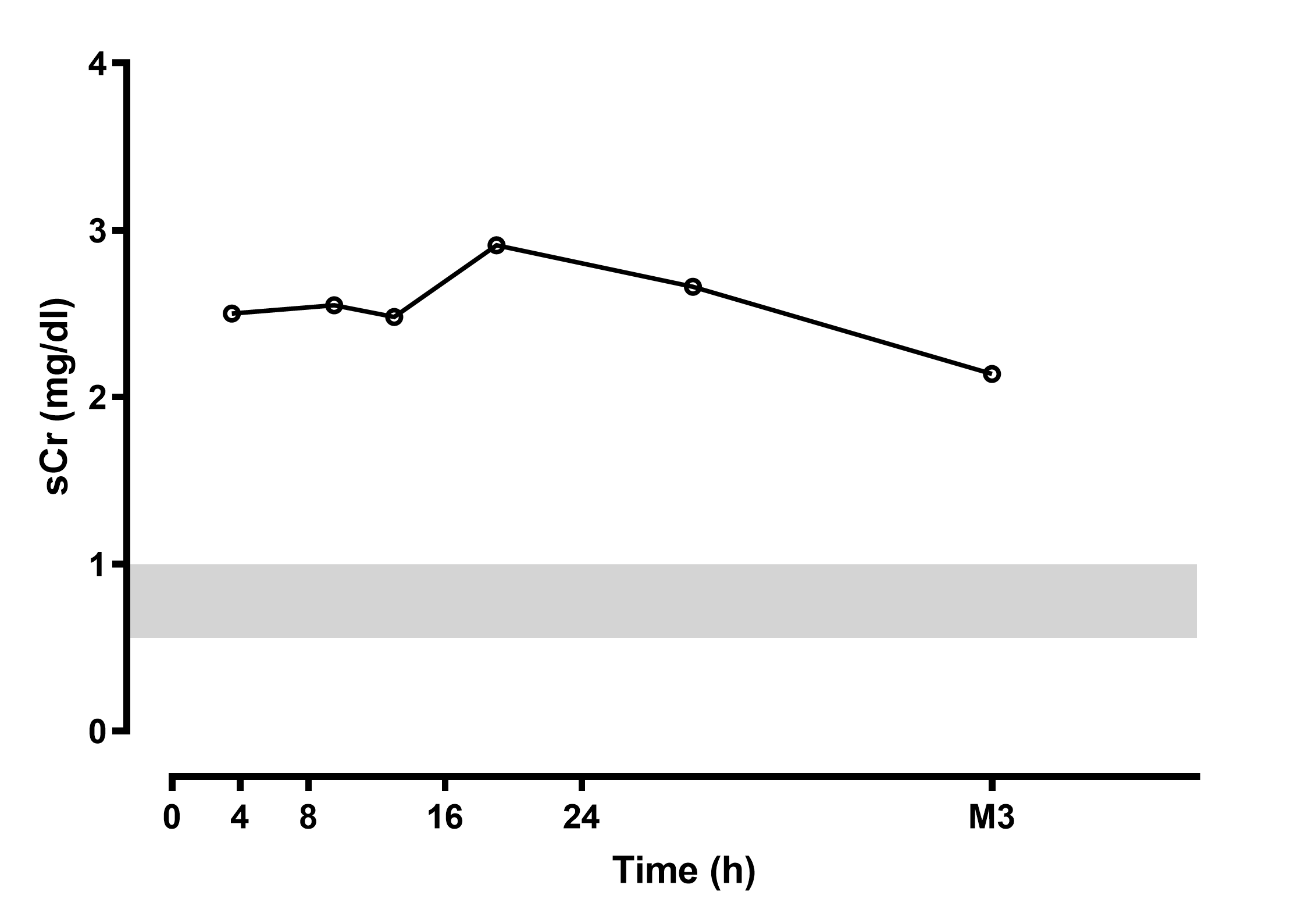

Supplement: S1 Fig — The grey shaded area is the normal range based on serum creatinine measured in healthy individuals. (TIF) [file pntd.0007486.s002.tif]

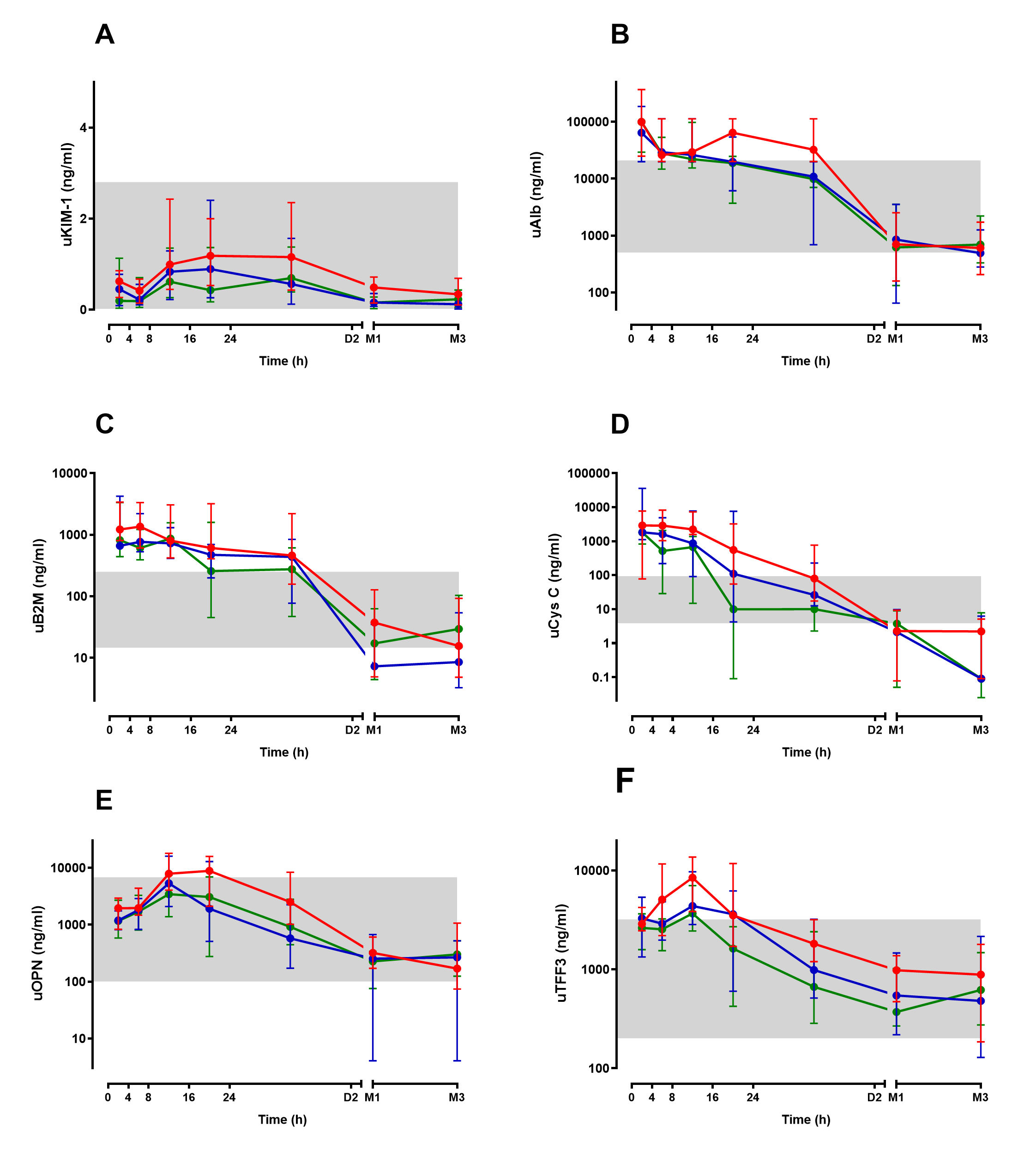

Supplement: S2 Fig — Time course of the median biomarker concentrations (± IQR) following Russell’s viper bite for 2 days then at 1 and 3 months, including urinary kidney injury molecule-1 (uKIM-1; Panel A), urinary albumin (uAlb; Panel B), urinary beta2-microglobulin (uβ2M; Panel C), urinary cystatin C (uCysC; Panel D), urinary osteopontin (uOPN; Panel E) and urinary trefoil factor-3 (uTFF3; Panel F). Patients without acute kidney injury (NO AKI; green), mild AKI (blue), moderate to severe AKI (red). The grey shaded area is the normal range based on respective biomarkers measured in healthy individuals. (TIF) [file pntd.0007486.s003.tif]

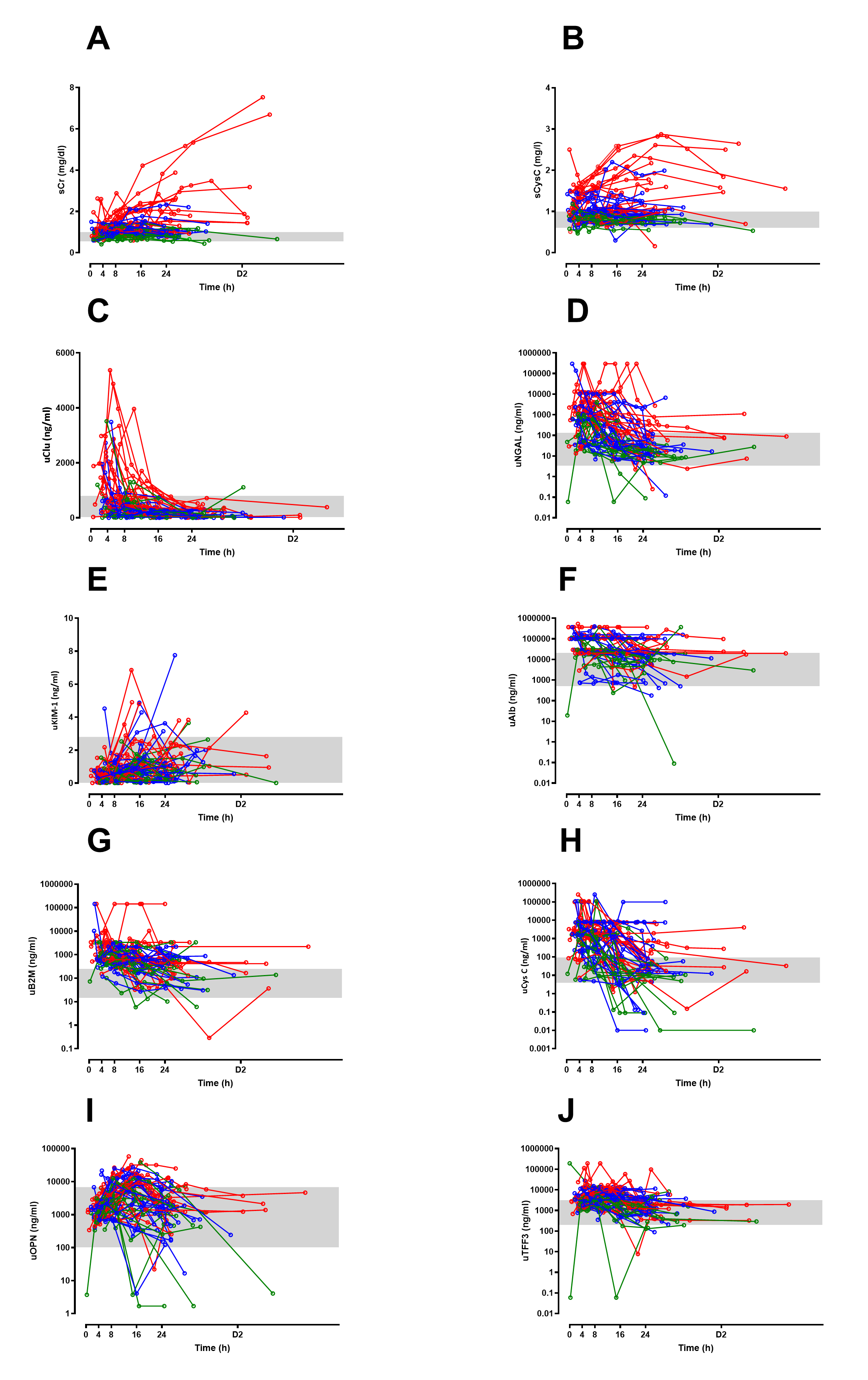

Supplement: S3 Fig — Absolute changes in each of the biomarkers following Russell’s viper bite for each individual patients over the first 2 days: [serum creatinine (sCr; Panel A), serum cystatin C (sCysC; Panel B), urinary clusterin (uClu; Panel C), urinary neutrophil gelatinase-associated lipocalin (uNGAL; Panel D), urinary kidney injury molecule-1 (uKIM-1; Panel E), urinary albumin (uAlb; Panel F), urinary beta2-microglobulin (uβ2M; Panel G),urinary cystatin C (uCysC; Panel H), urinary osteopontin (uOPN; Panel I) and urinary trefoil factor-3 (uTFF3; Panel J)]. Patients without acute kidney injury (No AKI; green), mild AKI (blue), moderate to severe AKI (red). The grey shaded area is the normal range based on respective biomarkers measured in healthy individuals. (TIF) [file pntd.0007486.s004.tif]

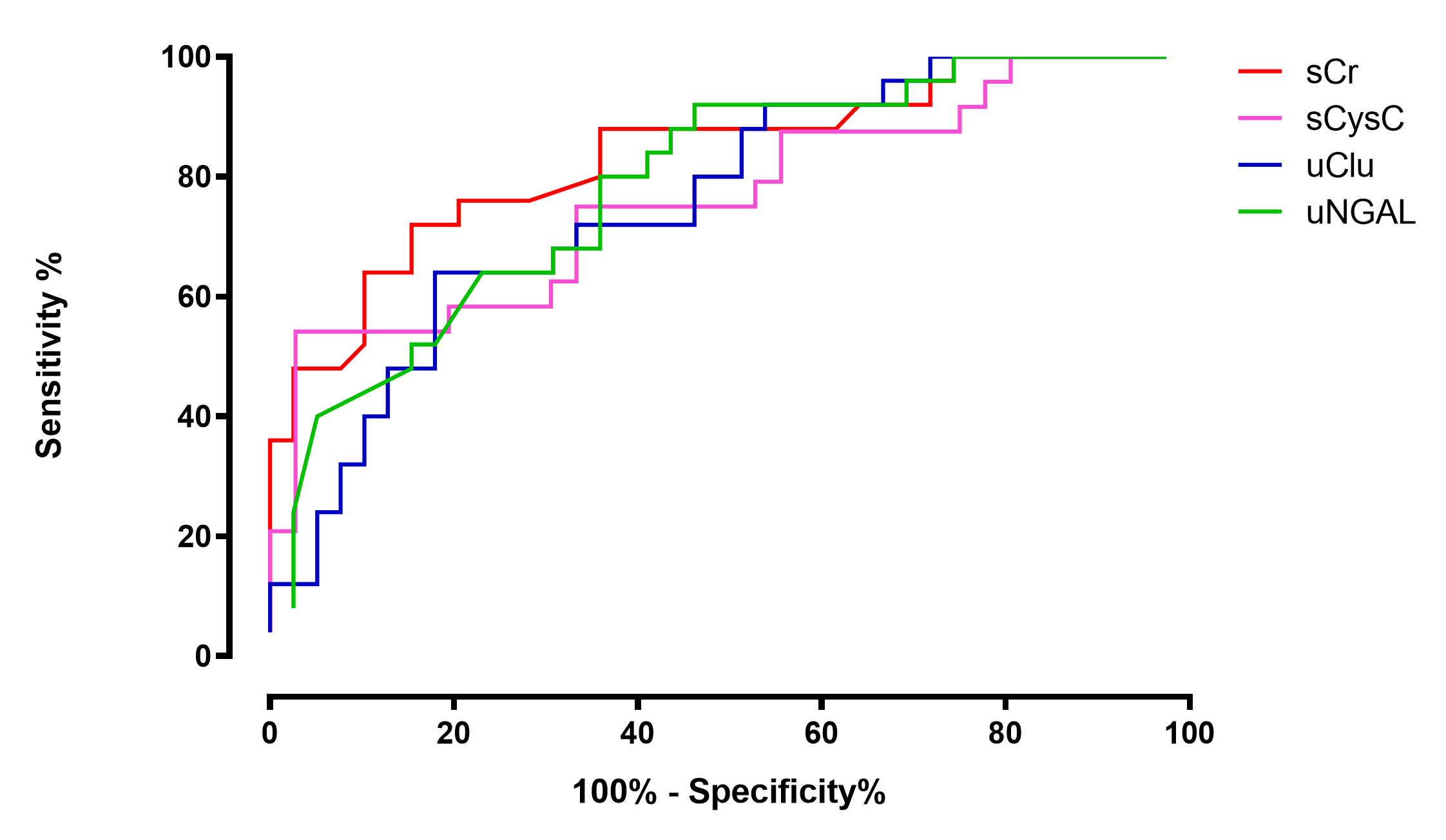

Supplement: S4 Fig — Receiver operator characteristic (ROC) curve analysis of the peak in each biomarker concentration within 24 hours post-bite detecting moderate to severe AKI versus No AKI/ mild AKI, for serum creatinine (sCr; red line), serum cystatin C (sCysC; pink line), urinary clusterin (uClu; blue line) and urinary neutrophil gelatinase-associated lipocalin (uNGAL; green line). (TIF) [file pntd.0007486.s005.tif]

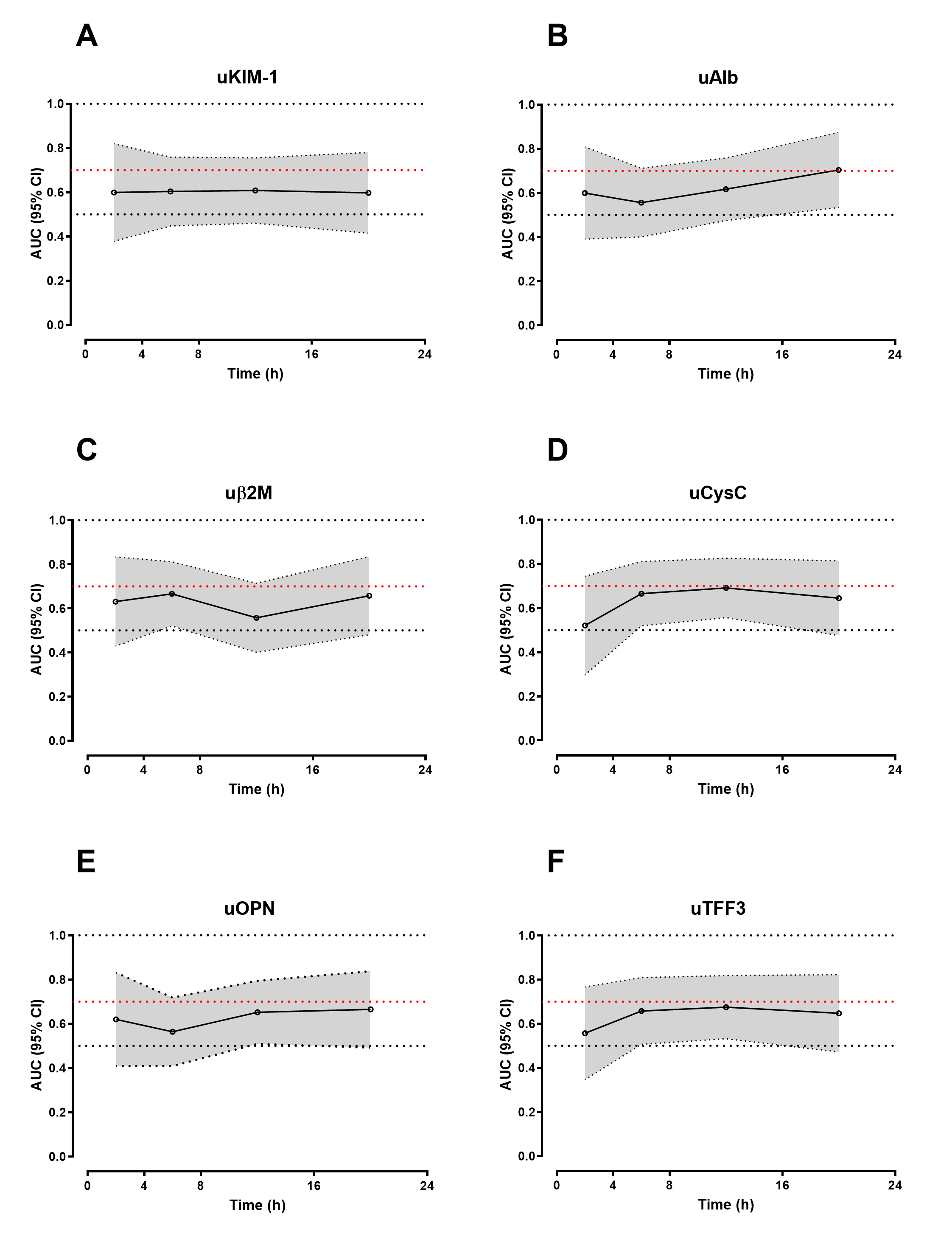

Supplement: S5 Fig — Plots of the AUC-ROCs versus time for six renal biomarkers in detecting moderate/severe AKI versus No AKI/ mild AKI, including urinary kidney injury molecule-1 (uKIM-1; Panel A),urinary albumin (uAlb; Panel B), urinary beta2-microglobulin (uβ2M; Panel C),urinary cystatin C (uCysC; Panel D), urinary osteopontin (uOPN; Panel E),and urinary trefoil factor-3 (uTFF3; Panel F). The dark black line represents the AUC-ROCs and the shaded area covers the 95% confidence intervals (CI) for the AUC-ROC. (TIF) [file pntd.0007486.s006.tif]
